# A Security Assessment Mechanism for Software-Defined Networking-Based Mobile Networks

**DOI:** 10.3390/s151229887

**Published:** 2015-12-17

**Authors:** Shibo Luo, Mianxiong Dong, Kaoru Ota, Jun Wu, Jianhua Li

**Affiliations:** 1 School of Electronic Information and Electrical Engineering, Shanghai Jiao Tong University, Shanghai 200041, China; luoshibo.pla@sjtu.edu.cn (S.L.); junwuhn@sjtu.edu.cn (J.W.); lijh888@sjtu.edu.cn (J.L.); 2 Department of Information and Electric Engineering, Muroran Institute of Technology, Muroran 050-8585, Japan; ota@csse.muroran-it.ac.jp

**Keywords:** 5G, security assessment, software-defined networking based mobile networks, attack graph, analytic hierarchy process

## Abstract

Software-Defined Networking-based Mobile Networks (SDN-MNs) are considered the future of 5G mobile network architecture. With the evolving cyber-attack threat, security assessments need to be performed in the network management. Due to the distinctive features of SDN-MNs, such as their dynamic nature and complexity, traditional network security assessment methodologies cannot be applied directly to SDN-MNs, and a novel security assessment methodology is needed. In this paper, an effective security assessment mechanism based on attack graphs and an Analytic Hierarchy Process (AHP) is proposed for SDN-MNs. Firstly, this paper discusses the security assessment problem of SDN-MNs and proposes a methodology using attack graphs and AHP. Secondly, to address the diversity and complexity of SDN-MNs, a novel attack graph definition and attack graph generation algorithm are proposed. In order to quantify security levels, the Node Minimal Effort (NME) is defined to quantify attack cost and derive system security levels based on NME. Thirdly, to calculate the NME of an attack graph that takes the dynamic factors of SDN-MN into consideration, we use AHP integrated with the Technique for Order Preference by Similarity to an Ideal Solution (TOPSIS) as the methodology. Finally, we offer a case study to validate the proposed methodology. The case study and evaluation show the advantages of the proposed security assessment mechanism.

## 1. Introduction

In recent years, Software-Defined Networking (SDN) has attracted great attention as an emerging future network architecture in fields such as 5G mobile networks, cloud services and so on. The most different thing about SDN compared to traditional network architectures is that its control plane is decoupled from the forwarding plane and the control plane is programmable. With these SDN features, the switches in SDN networks become simple forwarding devices. At the same time, the control plane is implemented in a logically centralized mode. All of these are helpful to simplify policy enforcement and make network configuration and evolution easy [1,2,3,4,5,6].

On the other hand, today’s mobile customers desire to remain connected anywhere, at any time, and using any device. This has triggered the investigation of 5G for the next generation of terrestrial mobile telecommunications. In this context, SDN-based Mobile Networks (SDN-MNs) have emerged as a future architecture for 5G [7,8,9,10,11]. SDN-MNs tend to connect all kinds of smart devices and interconnect other heterogeneous networks. More network devices, more types of network devices and more complex network connections are included in SDN-MN concept. This makes SDN-MNs more diverse and complicated than other networks.

Along with the benefits of SDN-MNs, the centralized control and programmability properties also introduce some new properties into the network with new security challenges. Because SDN-MNs provide the ability to directly program the whole network and to create dynamic flow policies instantly according to the current network context, the virtual property is introduced into SDN-MNs and their dynamic property is a consequence. Moreover, SDN has triggered significant interest in network function virtualization (NFV) [10]. NFV also brings programmability into its application networks. The programmability of SDN and NFV makes SDN-MNs more dynamic than traditional networks. Besides the dynamic nature brought by SDN and NFV, the services and endpoint equipment are mobile in 5G networks. The connectivity is also context-aware depending on the applications in 5G networks and is thus not predicable. All of the characteristics of 5G networks make SDN-MNs very dynamic.

The aforementioned dynamic nature and complexity of SDN-MNs lead to new challenges for security assessments in the SDN-MNs. To defend this type of network, the security methodology must address its dynamic nature and complexity. A number of security studies for SDN have recently been performed [12,13,14,15,16]. These works have found that the novel relationship among SDN elements in SDN networks introduces new vulnerabilities, and some of them are only present in SDN networks. For example, in the OpenFlow switch specification, Transport Layer Security (TLS) is used between the controllers and their switches with mutual authentication, but this security feature is not mandatory so it does not specify a standard for TLS. Benton *et al.* [14] found that OpenFlow is vulnerable to man-in-the-middle (MITM) attacks if TLS is not used, and due to its inherent centralized design property, OpenFlow is in the danger of Denial of Service (DoS). A high-level analysis of the overall security of SDN networks is discussed in [15]. They find that new threats are introduced and new response methods are needed, because of the inherent properties of the centralized design and programmability of SDN networks.

**Figure 1:**
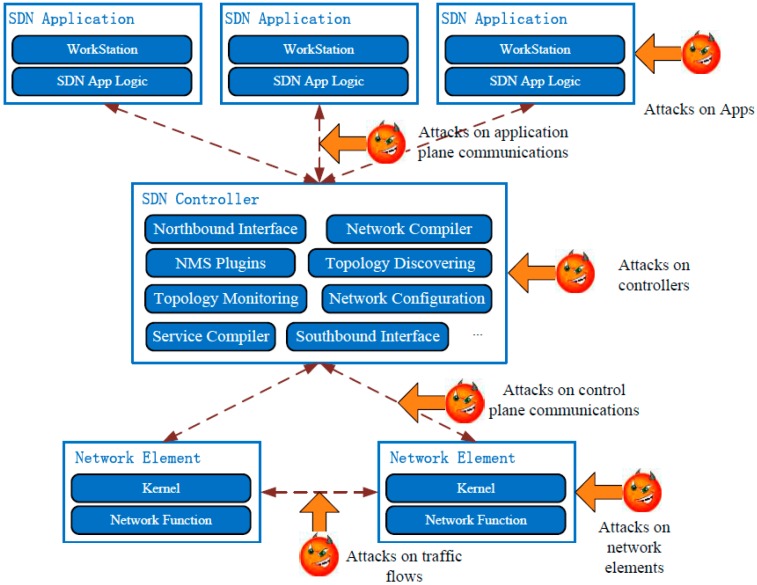
Security attack vectors of SDNs.

A comprehensive security attack vectors map of SDN is illustrated in Figure 1. Several attack vectors exist in applications, controllers, network elements and the links or traffic between them. Some of the attacks are common to all types of networks, such as the attack vectors on applications and network elements, but some of them only exist in SDN, such as the attack vectors on the SDN controller and the control links between the controller and network elements.

There is already lots of research focusing on traditional network security assessment. In the earlier period, security assessment methods were only applicable to isolated components. However as multi-stage attacks have become the most harmful network security threats, these methods are not enough. The reason is that these methods did not consider the security holes introduced by the interconnections of local vulnerability [17]. To address the problem of security holes, attack graphs have been proposed as security assessment methods by building security models of network systems [17,18,19,20,21]. In the research of Dantu *et al.* [22], attack graphs are used to model network vulnerabilities. Then they perform a risk analysis by a Bayesian method. The Bayesian method is also used to model potential attack paths in a system described by Liu and Man in [23]. Based on the background knowledge of the attacker, including attack mechanism, they develop algorithms to compute attack paths. Poolsappasit *et al.* [24] use a Bayesian method to quantify the chances of network compromise. Moreover, they develop a security mitigation and management plan according to these results.

However, none of them can be applied directly in SDN-MNs because they only focus on traditional networks and do not take the special properties of SDN-MNs into consideration. Firstly, these methods are only suitable for relatively static networks. They do not take the dynamic nature of SDN-MNs into consideration. Secondly, the traditional algorithms of attack graph generation are only suitable for relatively simple networks. When the network becomes more complex, the efficiency of these algorithms deceases greatly, so these traditional methods cannot deal with the diversity and complexity of SDN-MNs.

Based on the aforementioned analysis, it is very necessary to have an effective security assessment mechanism for SDN-MNs considering their distinctive features. Firstly, a methodology to measure the total security level of SDN-MNs is needed. When SDN-MNs interconnect nodes in the network, local vulnerabilities will introduce new security holes because of this connectivity [17]. This methodology needs to not only deal with the security holes, but also deal with the diversity and complexity of SDN-MNs. Secondly, a way to quantify the influence caused by dynamic properties of SDN-MNs is important as well, so besides the methodology, what factors and how they influence SDN-MN security assessments must be taken into consideration.

To address these problems, a security assessment scheme for SDN-MNs using attack graphs and an Analytic Hierarchy Process (AHP) is proposed. The rest of the paper is organized as follows: a background on mobile network architectures and SDN-MNs is introduced in Section 2. The structure of the proposed security assessment methodology is described in Section 3. Section 4 presents the details of the proposed attack graph model and the attack graph generation algorithm. The Node Minimal Effort (NME) attack graph quantification method considering the dynamic factors in SDN-MNs is discussed in Section 5. In Section 6, a case study is illustrated. Finally, we conclude the paper in Section 7.

## 2. Background

In the past few years, data traffic in mobile networks has seen an explosive growth. The Long Term Evolution (LTE) network architecture has been adopted to meet this evolution and nowadays the LTE architecture has been widely adopted by mobile service providers around the world [9,10,11,12,13].

**Figure 2:**
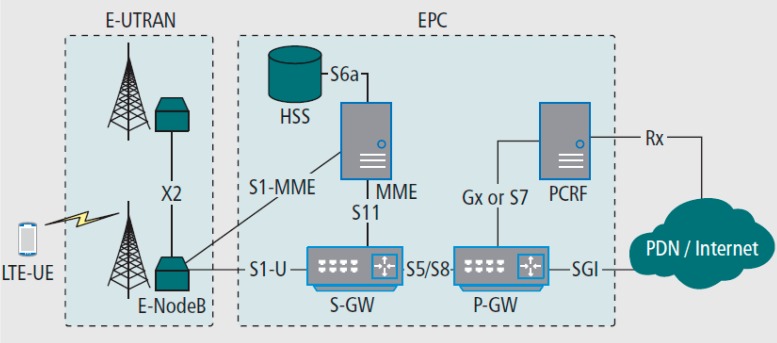
LTE network architecture.

Figure 2 illustrates the LTE architecture proposed by the 3rd Generation Partnership Project (3GPP). It is composed of the LTE core network called the evolved packet core (EPC) and Evolved Universal Terrestrial Radio Access Network (E-UTRAN). EPC includes the packet data network gateway (P-GW), the serving gateway (S-GW) and so on.

Although it significantly improves network performance, LTE creates some new problems. Because all traffic goes through the P-GW in EPC, the P-GW becomes a bottleneck to extend the mobile network, and because each device in this architecture uses specialized hardware and software, it greatly increases the time and equipment costs of the operators when introducing new network functionalities into LTE.

In order to address these challenges, many recent researches have proposed and discussed new mobile network architectures for 5G based on Software-Defined Networking [9,10,11,12,13].

**Figure 3:**
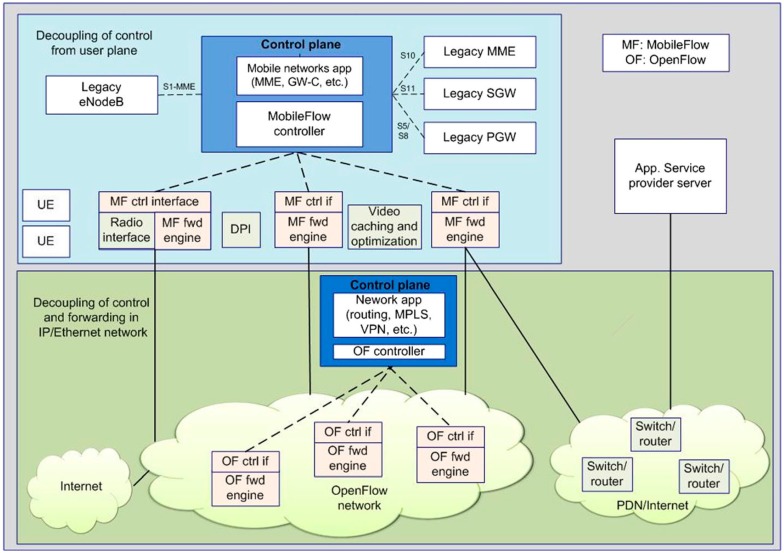
The MobileFlow network architecture.

For example, MobileFlow, illustrated in Figure 3, is proposed as a SDN-based mobile network architecture in [10]. The key components in the MobileFlow architecture are the MobileFlow controller and the MobileFlow forwarding engine. Similar to the SDN architecture, MobileFlow separates mobile network control from all user plane elements. MFFEs are interconnected by the IP network and are fully software driven. MobileFlow uses the OpenFlow protocol for communication between controllers and switches and support network layer tunneling. This makes MobileFlow much simpler than traditional EPS elements.

In [9], the authors propose a SDN-based architecture of mobile networks named Software-Defined Wireless Networks (SDWN). In the SDWN architecture, programmability is widely introduced into Radio Access Networks (RANs) and the core transport. An API is enabled in order to allow third parties to control the network.

In [13], the authors propose another SDN-based mobile network architecture named Cellular SDN (CSDN). In the CSDN architecture, radio access networks allow the orchestration of resources using the SDN and NFV techniques. Additionally, these techniques are leveraged for service orchestration.

## 3. Basics of Security Assessment Methodology

To provide a novel and complete methodology supporting the security assessment for SDN-MNs, there are two main questions that need to be considered. The first one is how to construct a complete security assessment methodology that can take the distinctive features of SDN-MNs into consideration. That means the methodology not only can take their dynamic nature into consideration, but also can promote the security assessment efficiency to address the complexity of SDN-MNs. The second one is how to quantify the security level of the network with regard to the various dynamic factors in SDN-MNs.

To construct the assessment methodology, we propose a novel attack graph modeling method to take the dynamic properties of SDN-MNs into consideration. To address the problem of attack graph scalability caused by the complexity of SDM-MNs, we propose a novel attack graph generation algorithm.

**Figure 4:**
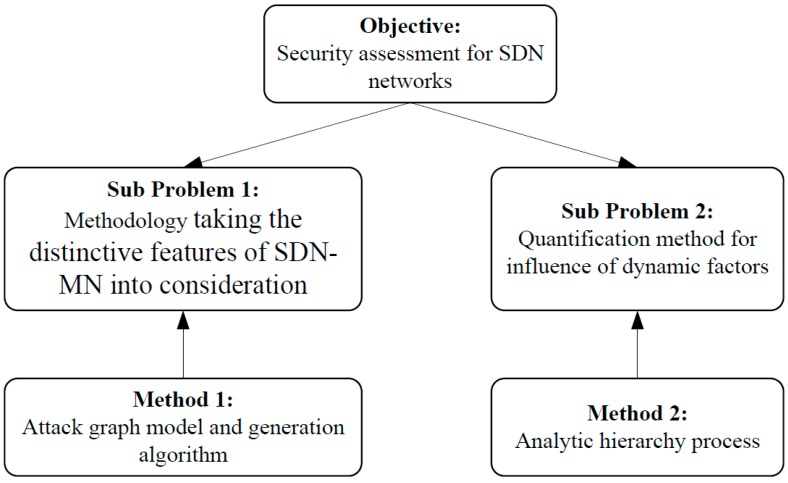
Basic idea of security assessment for SDN-MNs.

To quantify the security level of a SDN-MN, we define the NME that is used to derive the network security level. To calculate the NME with regard to the dynamic factors in an SDN-MN is a multiple criteria decision-making problem. We integrate AHP and the Technique for Order Preference by Similarity to an Ideal Solution (TOPSIS) to solve the problem. Expert knowledge is critical in the proposed methodology because AHP is a subjective method. The security assessment expert must know well the AHP and the SDN-MN itself. He or she can construct an AHP structure and appoint matrices according to the information collected from the network. The structure of the security assessment for SDN-MNs is illustrated in Figure 4.

## 4. Modeling Network Attack Using Attack Graph

An attack graph is defined as a collection of all scenarios of how an attacker achieves its goal to target a system [24,25]. As mentioned before, to represent multi-stage network attacks and generate attack graphs, lots of models and methods have been proposed. There is a common efficiency problem in these attack graph generation algorithms, in that SDN-MNs are often diverse and complicated, so an efficient attack graph generation algorithm is a must.

### 4.1. Definition of Attack Graph and Generation Algorithm

To define and generate attack graphs for SDN-MNs, we use previous work of our group which proposed an attack graph generation algorithm to address the efficiency problem [26]. 

The basic concepts of attack graph are defined as follows:

*Definition 1: Attack Graph*. An attack graph is defined as a tuple $\mathrm{AG}=(A,S,G,E_{b},E_{f})$. In the definition, A denotes Action objects, S denotes State objects and G denotes Goal objects. Also $E_{b}$ denotes backward pointers. Finally, $E_{f}$ denotes forward pointers.

**Figure 5:**
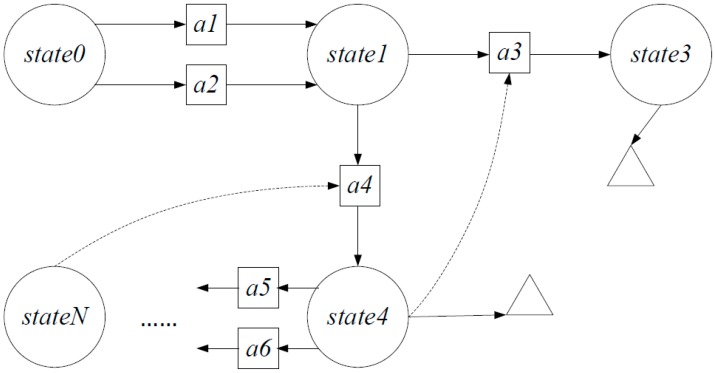
Attack graph definition for SDNs.

The attack graph definition is shown in Figure 5. In Figure 5, real lines denote forward pointers and dotted lines denote backward pointers. Rings identify the instances of State objects, and squares represent the instances of Action objects, and triangles represent the instances of Goal objects.

In the example, *state0* is the initial security state of the network. When action *a1* or *a2* is performed, the security state transfers to *state1*. Based on *state1*, if the attacker performs action *a3*, the security state continues to transfer to state3. At the end, the attacker reaches the attack goal. The rest of the attack graph has the same meaning.

**Figure 6:**
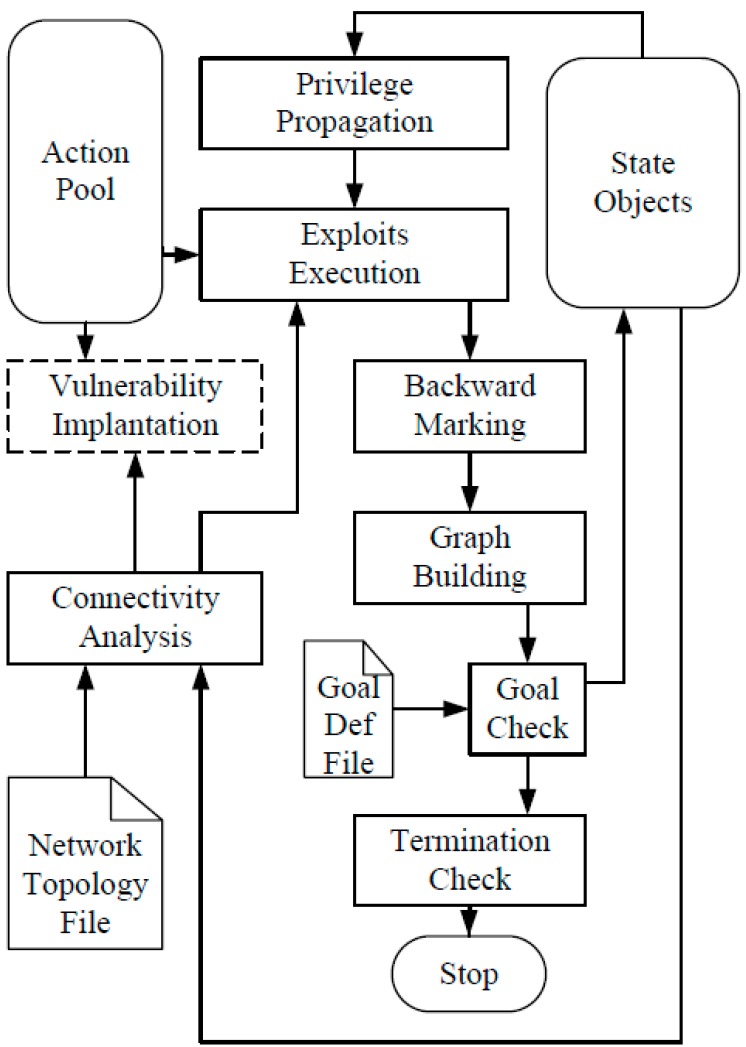
State evolution process for an attack graph.

According to the definition of attack graph given above, the attack graph generation algorithm generates attack graphs based on a network knowledge base. The network knowledge base includes vulnerabilities, network connectivity, *etc.* We use a “state evolution process” algorithm to generate attack graphs. The state evolution process is illustrated in Figure 6.

Until the process meets termination condition, state objects are recursively evolving in the process. In each cycle, the process applies all possible malicious actions. The process includes several function entities as follows:

(1) Privilege propagation entity

The privilege propagation function entity is responsible for propagate the privilege according to the current network state.

(2) Connectivity analysis entity

The connectivity analysis function entity firstly analyzes the network topology and filtering policy information and then it produces the connectivity between any two nodes.

(3) Exploits execution entity

The exploits execution function entity tries to apply all exploit objects to the state objects.

(4) Backward marking entity

The backward marking function entity removes all the former indicates. After actions are applied, it marks re-satisfied items with a fresh indicate for the consequences.

(5) Graph building entity

The graph building function entity generates a forward pointer or backward pointer according to the actions’ trigger.

(6) Goal check entity

The goal check function entity confirms whether the attack is successful in the current security context.

(7) Termination check entity

The termination check function entity confirms whether the process has no more ways to evolve. The process should be terminated once there is not a forward pointer. After a complete procedure, the generated attack graph is derived. For more detailed information about the sub-modules of the process readers can refer to [26].

### 4.2. Node Minimal Effort

Attack cost is the expense for an attacker to achieve an attack goal. We use attack cost in attack graphs to quantify the security level of the network. Namely, when the attack cost is higher, the network is more secure and *vice versa*. Normally, there are several actions in one attack behavior. We define action cost to designate the expense for an attacker to perform an action. To evaluate the attack cost of an attack path, we define NME for the nodes in the attack graph:

*Definition 2: In an attack graph, NME is the minimal attack cost to reach a node*.

There are two types of nodes in our attack graph definition, namely state node and action node. In general, the minimal effort of a state node is the attack cost of the attack path that leads to the state node. If there are several attack paths that leads to the state node, the minimal effort of the state is minimal attack cost in the attack paths. Because of the AND relation, the minimal effort of an action node equal all the NMEs of its prerequisite nodes plus the action cost of itself.

Let *min_effort* denotes NME, *min_effort* calculated as follows:

- for state node s∈S,

(1) $$\begin{array}{l} \text{min\_effort}(s)=0,\;\mathrm{if}s\;\text{is the initial state;}\\ \text{min\_effort}(s)=\min_{\text{k}}(\text{min\_effort}(a_{k})) \end{array}$$

where $\;a_{k}$ denotes a instance of the prerequisite nodes of s.

- for action node  a∈A,

(2) $$\text{min\_effort}(a)=\underset{k}{\sum }\text{min\_effort}(s_{k})+\text{effort}(a)$$

where $\;s_{k}$ denoted a instance of the prerequisite nodes of a, and effort(a) denotes the attack cost of a.

## 5. Quantification of NME Using AHP and TOPSIS

As mentioned before, the NME of a node equals to NMEs of its prerequisite nodes plus its own action cost, so the action cost is the basic variable and all the NMEs are derived from the action cost. The action cost is under the constraints of certain vulnerability and the dynamic factors of the network. This is a multiple criteria decision-making problem.

As AHP has been demonstrated to be an excellent multi-criteria decision-making approach in the past years, we adopt AHP to quantify the action cost and derive NME. To countermeasure the competitive benchmarking of the security properties for AHP, we integrate the technique for order preference by similarity to ideal solution (TOPSIS). This is because TOPSIS is an excellent compensatory aggregation method. We calculate action cost with the following steps:

Step 1: Construct the hierarchical security factors structure. In this step, we divide the hierarchical structure into four different layers.
Step 2: Calculate the weights of factors at each layer in the hierarchy using AHP. This step mainly includes deriving matrix data, calculating the weights of indicators, evaluating the consistency ratio and constructing combinatorial weights.
Step 3: Calculate the attack cost of actions in the dynamic environment using TOPSIS. This step mainly includes decision matrix normalization and so on. Finally, it calculates the attack cost of actions.

### 5.1. Construct Hierarchical Structure

**Figure 7:**
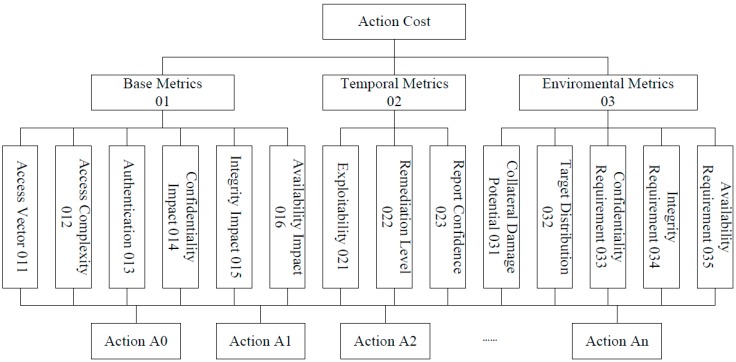
Hierarchical structure of security factors.

To quantify the attack cost of actions in a dynamic environment, we firstly need to construct the hierarchical structure of different security factors. We divide the hierarchical structure into four different layers in this paper. Firstly, the top layer is the goal layer. In our paper, the goal is to determine the action cost. Below the top layer, there is the criteria layer. This layer includes the main types of metrics groups for security assessment. Below the criteria layer, there is the indicator layer. In this layer, we subdivide the metrics group into single metrics and we construct the action layer as the forth layer. In this layer, actions involved in the attack graph are included. We use the Common Vulnerability Scoring System (CVSS) as the basic for the structure [27,28]. Figure 7 shows the hierarchy.

### 5.2. Calculate the Weights of Factors Using AHP

Pair-wise comparison is the basis of the AHP analysis [29]. We use the pair-wise comparison scale for AHP preferences as in [29].

#### 5.2.1. Matrix Data

Once the hierarchy has been constructed, we determine the pair-wise comparison matrix. According to the AHP method, we first construct the pair-wise comparison matrix *A*:

(3) $$A={\left(a_{ij}\right)}_{n\times n}\text{=}\left[\begin{array}{c} \begin{array}{cc} 1 & a_{12}\\ a_{21} & 1 \end{array}\begin{array}{cc} \ldots & a_{1n}\\ \ldots & a_{2n} \end{array}\\ \begin{array}{cc} \ldots & \ldots \\ a_{n1} & a_{n2} \end{array}\begin{array}{cc} \;\ldots & \ldots \\ \ldots & 1 \end{array} \end{array}\right]$$

In matrix *A*, the parameter $a_{ij}$ represents the relative importance of the ith factor compared to the jth factor, and aij=1aji.

#### 5.2.2. Weights of Indicators

The indicator weight can be derived from the relevant normalized eigenvector $W=(w_{1},w_{2},\ldots ,w_{n})$ of corresponding matrix *A* as follows:

(4) $$AW=\lambda_{\max}W$$

In the equation, $\lambda_{max}$ represents the largest eigenvalue of matrix *A*.

#### 5.2.3. Consistency Ratio

We use the following steps to evaluate the consistency ratio (CR) of the estimated vectors:

Step 1: Calculate the eigenvector and $\lambda_{max}$ for each matrix.

Step 2: Compute the consistency index (*CI*) as follows:

(5) $$CI=(\lambda_{max}-n)/(n-1)$$

Step 3: Calculate *CR* as follows:

(6) CR=CI/RI

*RI* is a known random consistency index. It is derived from the average index of randomly generated weights. A *CR* value must be less than 0.10. Otherwise, we must reapportion the parameters of matrix *A*.

#### 5.2.4. Construct Combinatorial Weights

The last step in AHP procedure is to construct the combinatorial weight:

(7) $$W^{\left(i\right)}=(W_{1}^{\left(ip\right)},W_{2}^{\left(ip\right)},\ldots ,W_{n}^{\left(ip\right)})W^{\left(p\right)}$$

In the equation, $W^{\left(p\right)}$ represents the criteria layer combinational weight vector, and $\left(W_{1}^{\left(ip\right)},W_{2}^{\left(ip\right)},\ldots ,W_{n}^{\left(ip\right)}\right)$ represents the indicator layer weight matrix.

### 5.3. Calculate the Attack Cost of Actions using TOPSIS

After constructing the matrix *A*, the values of the vulnerability factors should be standardized and the attack cost of actions in the dynamic environment is calculated. Both of them use the TOPSIS method.

#### 5.3.1. Normalize Decision Matrix

The matrix *A* is normalized to form the normalized decision matrix *T*:

(8) $$T={\left(T_{ij}\right)}_{m\times n}$$

The normalization method is as follows:

(9) $$t_{ij}=a_{ij}/\sqrt{\overset{n}{\underset{j=1}{\sum }}a_{ij}^{2}},i=1,2,\ldots ,m,j=1,2,\ldots ,n$$

#### 5.3.2. Compute the Weighted Normalized Decision Matrix and Alternatives

The weighted normalized decision matrix *F* is derived as follows:

(10) F=TW

In the equation, W represents a diagonal matrix. The matrix is composed by the weight in $W^{\left(i\right)}$.

Define the best alternative $V^{+}$ and the worst alternative $V^{-}$:

(11) $$V^{+}=\left(v_{1}^{+},v_{2}^{+},\ldots ,v_{n}^{+}\right)$$

(12) $$V^{-}=\left(v_{1}^{-},v_{2}^{-},\ldots ,v_{n}^{-}\right)$$

Let *I* denotes the benefit criteria, and *J* denotes the cost criteria. Then the values of $V^{+}$ and $V^{-}$ can be calculated as follows:

(13) $$v_{i}^{+}=\{\max(f_{ij})|i\in I\},\{\min(f_{ij})|i\in J\}$$

(14) $$v_{i}^{-}=\{\min(f_{ij})|i\in I\},\{\max(f_{ij})|i\in J\}$$

#### 5.3.3. Calculate the Attack Cost of Actions

Derive the L2-distance between alternative *i* and $V^{+}$ as follows:

(15) $$s_{i}^{+}=\sqrt{\overset{n}{\underset{j=1}{\sum }}{\left(v_{ij}-v_{j}^{+}\right)}^{2}}$$

Also, derive the L2-distance between alternative *i* and $V^{-}$ as follows:

(16) $$s_{i}^{-}=\sqrt{\overset{n}{\underset{j=1}{\sum }}{\left(v_{ij}-v_{j}^{-}\right)}^{2}}$$

Based on the distances, we calculate the attack costs of actions by the following formula:

(17) $${\mathrm{cost}}_{i}=\frac{s_{i}^{+}}{s_{i}^{+}+s_{i}^{-}},i=1,2\ldots ,m$$

## 6. Case Study

### 6.1. Case Network

**Figure 8:**
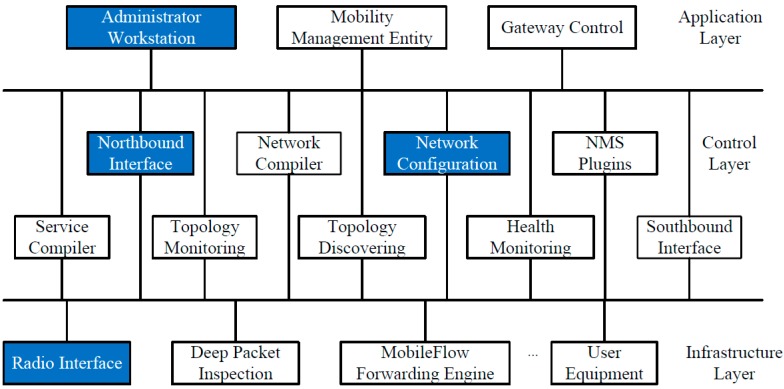
Case of Software-Defined mobile network.

We provide a SDN-MN case based on the MobileFlow architecture [10] and Kumar's solution [30] as an example of a security assessment. Based on [10], we construct the whole network layers and some main network components special to SDN-MN. Based on [30], we detail the components of the MobileFlow controller. As shown in Figure 8, the network is divided into three layers. The application layer includes various applications utilizing the SDN-MN controller to manipulate the network infrastructure. The control layer includes the SDN-MN controller components, such as application interface, service compiler, network compiler, *etc*. The infrastructure layer includes the radio interface, deep packet inspection, MobileFlow forwarding engine, user equipment, *etc*.

We suppose some of the function modules in the network are vulnerable. The vulnerable modules include the “Administrator Workstation” module, “Northbound Interface” module, “Network Configuration” module, and “Radio Interface” module. The vulnerabilities and their details are listed in Table 1.

**Table 1:** Details of vulnerabilities in case network.

ID	Node Name	CVE#	Detail
1	“Administrator Workstation” module	CVE-2004-0330	Vulnerability that allows remote users to execute arbitrary code in some Serv-U versions.
		CVE-2004-1992	Vulnerability that allows remote attackers to execute DoS in some Serv-U versions.
2	“Northbound Interface” module	CVE-2003-0533	Stack buffer overflow in Active Directory service.
3	“Radio Interface” module	CVE-2004-0417	Integer overflow in some CVS versions.
		CVE-2004-0415	Vulnerability that allows local users to access portions of kernel memory.
4	“Network Configuration” module	CVE-2002-0392	Vulnerability that allows remote attackers to execute DoS and execute arbitrary codes.

To explain the influence toward the security assessment results of dynamic factors, we define two schemes. The two schemes differ in the decision matrix. This means certain actions affect the security differently in different environments.

### 6.2. Security Model for Analysis

**Figure 9:**
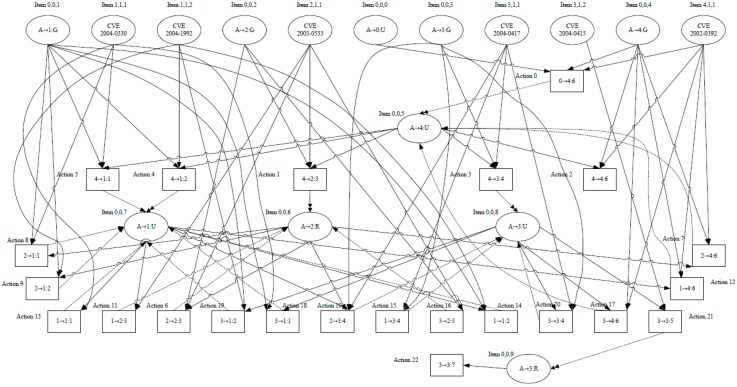
Generated attack graph case.

We suppose that the attacker wants to hack the “Radio Interface” module and then ultimately disable the “Radio Interface” module, that is, the “Radio Interface” module is the ultimate target of the attacker. We generate the attack graph to attack the “Radio Interface” module as shown in Figure 9 using the proposed attack graph generation algorithm.

In Figure 9, a circle denotes the State object labeled “Item x, y, z”. The number x denotes the object ID in Table 1. The number y denotes the type of object. The number z denotes the instance ID of the object, and the text in the circle is a description of the item. A square denotes an Action object and other components are defined in Definition 1.

There are in total 23 actions in the attack graph.

### 6.3. Calculate the Values of the Actions Costs

In this section, we collect data of the AHP matrices. In our case, the AHP matrices are illustrated in Table 2, Table 3, Table 4 and Table 5. The decision matrixes of actions compared to the indicators of the two schemes are listed in Table 6. We call the first scheme the “Network Administrator” scheme. In this scheme, to provide the network administrator convenience to manage the network, the “Network Configuration” module is accessed easily. We call the second scheme the “Application User” scheme. In this scheme, to provide the application user convenience to call the Northbound API, and the “Northbound Interface” module is accessed easily.

**Table 2:** AHP matrix of the criteria layer.

	01	02	03
01	1	1/3	1/5
02	3	1	1/2
03	5	2	1

**Table 3:** No. 01 AHP matrix of the indicator layer.

	011	012	013	014	015	016
011	1	1	1/3	1/3	1/2	1/2
012	1	1	1/3	1/3	1/2	1/2
013	3	3	1	1	1/2	1/2
014	3	3	1	1	1/2	1/2
015	2	2	2	2	1	1
016	2	2	2	2	1	1

**Table 4:** No. 02 AHP matrix of the indicator layer.

	021	022	023
021	1	2	1/2
022	1/2	1	1/3
023	2	3	1

**Table 5:** No. 03 AHP matrix of the indicator layer.

	031	032	033	034	035
031	1	1/3	3	1	2
032	3	1	6	3	5
033	1/3	1/6	1	1/2	1
034	1	1/3	2	1	2
035	1/2	1/5	1	1/2	1

**Table 6:** Decision matrix in our case.

	“Network Administrator” Scheme	“Application User” Scheme
011	13113511131135135111131	15553955155539553955515
012	13223522132235225221132	15663966156639663966615
013	13224622132246224622113	15664966156649664966615
014	34334633343346334633134	37774774377747744777437
015	33443643334436433643133	37773773377737733777337
016	87515511875155115511187	82515211825152115211182
021	36346841363468416841136	47457952474579527955247
022	36346841363468416841136	36346841363468416841136
023	25135631251356315631125	58568962585689628962258
031	59119911591199119911159	59119911591199119911159
032	11117111111171117111111	11777171117771717171111
033	73556353735563536353373	73556353735563536353373
034	75776573757765736573375	75776573757765736573375
035	75556553755565536553375	75556553755565536553375

**Figure 10:**
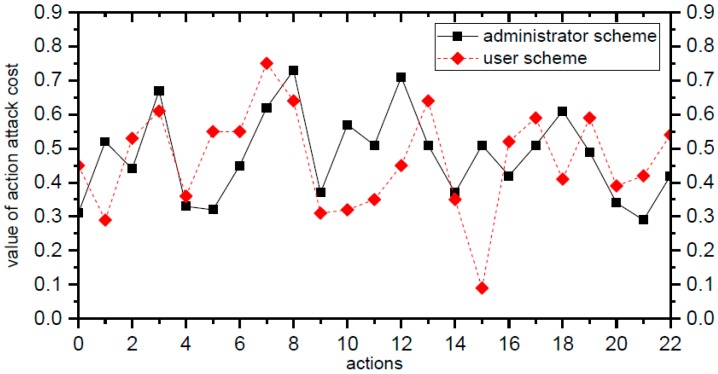
Attack cost values of actions.

Finally, we calculate the values of the attack costs of actions of the two schemes using the formulas in Section 4. The result is shown in Figure 10.

### 6.4. Calculate the Minimal Effort and Get the Shortest Attack Path

**Figure 11:**
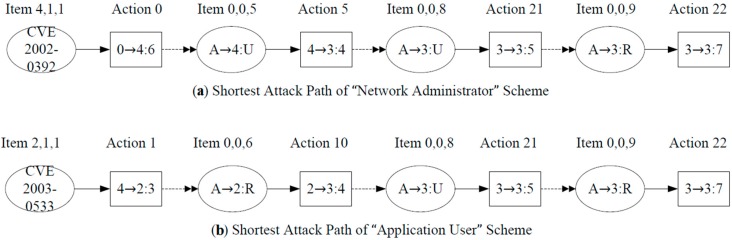
Shortest attack paths: (**a**) “Network Administrator” scheme; (**b**) “Application User” scheme.

Based on the values of the actions attack costs and the definition of NME, we can draw the two shortest attack paths of the schemes and the corresponding minimal efforts separately for the “Radio Interface” module. Figure 11 shows the shortest attack paths of the two schemes separately.

In both schemes, action 22 is the last node of the corresponding attack paths, so the NME of action 22 can designate the security level of the network.

According to the action attack cost and *min_effort* equation values, we can calculate the minimal effort of action 22 in the two schemes separately. Let *min_effort1* denote the *NME* value in the “Network Administrator” scheme and *min_effort2* denote the *NME* value in the “Application User” scheme. In addition, let $cost\;\left(a_{i}\right)$ denote the cost of the *i*th action. Then, the values are calculated as follows:

(18) $$\mathrm{min\_effort}1=\underset{i=0,5,21,22}{\sum }\mathrm{cost}(a_{i})=1.34$$

(19) $$\mathrm{min\_effort}2=\underset{i=1,10,21,22}{\sum }\mathrm{cost}(a_{i})=1.56$$

The *NME* of action 22 in the “Network Administrator” scheme is 1.34, and the *NME* of action 22 in the “Application User” scheme is 1.56. Intuitively, the cost of exploitation of CVE2002-0392 is less expensive than CVE2003–0533 in the “Network Administrator” scheme, so in the “Network Administrator” scheme the attack is launched from the “Network Configuration” module easier than from other modules. However, in the “Application User” scheme, the cost of exploitation of CVE2002-0392 is more expensive than CVE2003–0533 because of the change of the network factors, so the initial node of attack is the “Northbound Interface” module. From the example, we can learn that when the network factors vary, the minimal effort of action 22 varies.

## 7. Conclusions and Future Work

SDN-MNs provide many advantages for 5G mobile networks, but they also introduce new security challenges into the networks. The main problem is how to measure the security level of SDN-MNs. In this paper, we have proposed a novel security assessment mechanism for SDN-MNs. We analyze the new characteristics of SDN-MNs and find that SDN-MNs introduce a dynamic nature and complexity into mobile networks. The traditional network security assessment mechanisms cannot be directly applied to SDN-MNs. To solve the new security problems, we propose a security assessment methodology based on attack graphs and an analytic hierarchy process. A novel definition and attack graph generation algorithm are proposed to address the complexity of SDN-MNs. Also, we define NME as the index value of a security assessment to capture the quantitative dynamic property influence of SDN-MNs. By evaluating our scheme, we prove that our security assessment mechanism can deal precisely with the distinctive features of SDN-MNs and is very effective for security assessment for SDN-MNs. However, we use the classical AHP in our mechanism, so there are still margins for improvement. The classical AHP has the property of unbalanced scale of judgment. In the pair-wise comparison process, it is not precise to solve the inherent uncertainty and vagueness. In the future, it would be useful to employ a fuzzy AHP approach [31]. With a fuzzy AHP approach, quantifications of attack costs would become more precise.
